# Evaluation of Four Clinical Metrology Instruments for the Assessment of Osteoarthritis in Dogs

**DOI:** 10.3390/ani12202808

**Published:** 2022-10-17

**Authors:** João C. Alves, Ana Santos, Patrícia Jorge, Catarina Lavrador, Luís Miguel Carreira

**Affiliations:** 1 Divisão de Medicina Veterinária, Guarda Nacional Republicana (GNR), Rua Presidente Arriaga, 9, 1200-771 Lisbon, Portugal; 2 MED—Mediterranean Institute for Agriculture, Environment and Development, Instituto de Investigação e Formação Avançada, Universidade de Évora, Pólo da Mitra, Ap. 94, 7006-554 Evora, Portugal; 3 Faculty of Veterinary Medicine, University of Lisbon (FMV/ULisboa)—Portugal, 1300-477 Lisbon, Portugal; 4 Interdisciplinary Centre for Research in Animal Health (CIISA), University of Lisbon (FMV/ULisboa)—Portugal, 1300-477 Lisbon, Portugal; 5 Anjos of Assis Veterinary Medicine Centre (CMVAA), 2830-077 Barreiro, Portugal

**Keywords:** animal model, dog, osteoarthritis, hip, stance analysis, clinical metrology instruments

## Abstract

**Simple Summary:**

Osteoarthritis is a very common joint disease in dogs. For that reason, it is important to have validated owner questionnaires to assess patients and response to eventual treatments. For dogs, several questionnaires exist, with a growing interest on their validation. We aimed to evaluate four of them and determine their ability to evaluate pain and patient outcome. The questionnaires we evaluated can be used for the evaluation of osteoarthritis and response to treatment in dogs.

**Abstract:**

Osteoarthritis (OA) is the most commonly diagnosed joint disease in companion animals, and proper tools are necessary to assess patients and response to treatment. We aimed to perform the psychometric evaluation of several clinical metrology instruments (CMI), developed to evaluate pain and assess outcome. Fifty police working dogs with bilateral hip OA were assessed in a prospective, randomised, double-blinded study. Patients were evaluated using a stance analyser in six different moments divided over a 180-day period. Pedometer step count, weight-bearing symmetry index and deviation from normal weight-bearing were calculated and used for criterion validity. In each evaluation moment, a copy of the Hudson Visual Analogue Scale (HVAS), Canine Brief Pain Inventory (CBPI), Liverpool Osteoarthritis in Dogs (LOAD) and Canine Orthopaedic Index (COI) were completed by the dogs’ handlers. Correlations between CMIs were evaluated as construct validity. Further evaluation was performed with the Kaiser–Meyer–Olin measure of sampling adequacy, Eigenvalue and scree-plot analysis. Internal consistency was tested with Cronbach’s α. Significant weak correlation was found between all CMIs and stance analysis symmetry index measure and deviation, indicating criterion validity. Significant weak correlation was also found between pedometer count and LOAD plus COI. Cronbach’s α was 0.80 for HVAS, 0.98 for CBPI, 0.97 for LOAD and 0.98 for COI. Significant strong correlation was observed between CMIs, indicating construct validity. We present criterion and construct validity of these CMIs, which are able to capture various dimensions of OA. They can be used for the evaluation of osteoarthritis and response to treatment in dogs.

## 1. Introduction

Osteoarthritis (OA) affects all mammals, being an important and costly disease in humans and dogs [1,2]. It is the most commonly diagnosed joint disease both in human and veterinary medicine and represents a significant burden to societies, as it affects quality of life and performance and implies a large cost in terms of healthcare, posing major welfare challenges and concern [3,4]. The pathologic process, clinical presentation and response to treatment are very similar in humans and dogs, making the dog a frequent animal model for the study of OA [5]. This model provides an anatomical resemblance and similar disease progression as well as same shared environment and lifestyle, with the advantage of providing a faster disease progression, thus making it easier to study. For these reasons, study results can be translated to human OA [5,6,7,8,9,10]. However, this model has its limitations, as the dog has a quadruped stance rather than a bipedal one, is frequently sexually altered at an early age, and dog OA can be secondary to a developmental disease [5,6]. Still, exploring spontaneous dog OA under the One Medicine initiative can help improve health and well-being of both humans and dogs [5].

Pain is the most relevant clinical sign of OA and a hallmark of the disease [3,11]. It is a multi-dimensional experience that has sensory, evaluative and affective components [12]. Since it is central in both human and veterinary clinical practice, the current therapeutic goal for both is the management of pain and associated loss of function [13,14]. Measuring and evaluating pain is key to assess relevance and utility of any specific animal model on translation research [11,12]. Several clinical metrology instruments (CMI) have been developed in order to evaluate pain and assess outcome. As a whole, they show discrimination, responsiveness and criterion validity as measures of pain and impairment in performing daily activities and represent a patient-centred approach that, similar to what happens in human medicine, has been incorporated in veterinary assessments [5,15,16]. Two of the most widely used are the Canine Brief Pain Inventory (CBPI) and the Liverpool Osteoarthritis in Dogs (LOAD) [17,18,19,20]. The CBPI encompasses two sections, a pain severity score (PSS) and a pain interference score (PIS). The first assesses the magnitude of pain of the animal and the second assesses the degree to which pain affects daily activities [21]. The Canine Orthopaedic Index (COI) was developed to assess four dimensions of OA in dogs: stiffness, gait, function and quality of life [22]. The Hudson Visual Analogue Scale (HVAS) has been compared with force plate analysis, shown to be repeatable and valid for assessing the degree of mild to moderate lameness [23].

Mobility is important to overall health and wellbeing. Mobility impairment and decreased activity are associated with musculoskeletal pain in humans, and improved results in regards to mobility have been recommended as measures of outcome [17]. There are several means for quantitatively analysing mobility impairment. Ground reaction forces (GRF) have been described as outcome measures reflecting pain-related functional impairments in the context of OA, being abnormally lower in dogs with OA [24,25,26]. Weight distribution and off-loading or limb favouring at the stance are commonly used subjective assessments during orthopaedic examination [27]. Patients with OA may not be overtly lame at a walk or trot but exhibit subtle shifts in body weight distribution at a stance due to pain or instability [28,29]. Stance analysis has been reported as sensitive for detecting lameness in dogs [30]. It has been proposed that body weight distribution at a stance may, in fact, be an equivalent or superior measurement of pain associated with hip OA than both vertical impulse and peak vertical force (PVF) [29,31]. Pedometers are inexpensive, simple devices that measure ambulatory activity with acceptable accuracy [32].

The aim of this study was to compare HVAS, CBPI, LOAD and COI with each other to evaluate construct validity. We also aimed to compare these CMIs to objective measures (stance analysis and pedometer step counts) to test criterion validity and compare changes in a follow-up study. In addition, we wanted to test factor analysis of the CMIs. We hypothesised that correlation would be found between the four considered CMIs, and also between them and the considered objective measures.

## 2. Materials and Methods

The study protocol was approved by the ethical review committee of the University of Évora (Órgão Responsável pelo Bem-estar dos Animais da Universidade de Évora, approval nº GD/32055/2018/P1, 25 September 2018), and complies with relevant institutional, national and ARRIVE guidelines for the care and use of animals. Written, informed consent was obtained from the institution responsible for the animals. A sample of 50 police working dogs (N = 50) of both sexes was used, a convenience sample selected for a longitudinal double-blinded, negative-controlled study evaluating intra-articular treatment modalities in a naturally occurring canine OA model. Data from this study were used for the present analysis, with information collected at the initial diagnostics evaluation being used as a cross-sectional cohort while follow-up evaluations assessing response to treatment constituted the longitudinal cohort. Inclusion and exclusion criteria were defined by research activity other than that currently reported and are summarised in Table 1. Dogs in this population were not spayed or neutered and were screened for hip and elbow dysplasia before beginning training.

The study was conducted over a period of 180 days, and data were gathered on days 0, 8, 15, 30, 90 and 180. On day 0 (treatment day), patients either received an intra-articular administration of 0.9% NaCl (control group) or a treatment (a platelet concentrate—V-PET^®^, hylan G-F 20, triamcinolone hexacetonide or stanozolol), according to the assigned group. All groups had the same number of animals. No other medications/treatments were administered during the follow-up period. In all evaluation moments, a copy of all CMIs was completed by the handlers, stance analysis was performed, and pedometer count was recorded.

Before completion of an online copy of the HVAS, CBPI, COI and LOAD, handlers received the published instructions for each of them. The CMIs were completed in sequence by the same handler in each of the follow-up assessments, without knowledge of their previous answer, in a calm room with as much time as needed to answer all items. As CBPI has two sections (PSS and PIS) and COI has four dimensions (stiffness, function, gait and QOL), we considered all sections and dimensions in the analysis [33]. Handlers were unaware of both the stance analysis and step count results before completing the CMIs.

Stance analysis was conducted with a weight distribution platform (Companion Stance Analyzer; LiteCure LLC^®^, Newark, DE, USA). According to manufacturer’s guidelines, the platform was placed in the centre of a room, at least 1 m from the walls, and calibrated at the beginning of each day and zeroed before each data collection. After a period of familiarisation with the space, animals were encouraged by their handlers to stand on the weight distribution platform with one foot on each quadrant of the platform while maintaining a natural stance with their centre of gravity and stability (measured by the platform) near the middle of the platform. Gentle restraint was used to maintain the patient’s head in a natural, forward-facing position. For all animals, at least 20 measurements were performed, and the mean value was determined. Evaluations were conducted in the morning, and no exercise or activity was performed prior to it, as exercise can exacerbate pelvic limb lameness and is a potential factor of variation during gait evaluation [34]. Normal weight distribution for the pelvic limbs was considered 40% (20% right pelvic limb + 20% left pelvic limb) of the total weight [29].

Pedometers (Xiaomi wrist pedometer) were worn around the dog’s neck, attached to an adjustable lightweight collar, so that they detected and counted forelimb steps only, associated with greater accuracy at a walk, trot or run [35]. They were placed one week before the first evaluation moment in order to determine a baseline value and then maintained up to the 30th day post-treatment. For the 90th and 180th post-treatment days evaluation, the animals wore the pedometer for a week before that evaluation moment. The mean daily counts were considered and calculated by dividing the registered number of steps by the number of days considered.

To test criterion validity, CMI scores were compared with the left–right symmetry index (SI), calculated with the following formula: SI = [(WBR − WBL)/((WBR + WBL) × 0.5)] × 100 [19,36,37], where WBR is the value of weight bearing for the right pelvic limb and WBL is the value of weight bearing for the left pelvic limb. Negative values were made positive. As normal weight bearing for the pelvic limbs is 40%, we also considered deviations from this value, obtained by subtracting WBR + WBL to 40. To additionally test validity, we compared CMI scores against changes in activity, considered as changes in mean daily step count with the pedometers.

We compared the results of the CMIs using repeated measures of ANOVA with a Huynh–Feldt correction and tested construct validity by comparing individual CMI scores against all others. Correlation was assessed with Spearman’s rank correlation coefficient, with *p* < 0.05. Additionally, we performed factor analysis for all CMIs using the Kaiser–Meyer–Olin (KMO) measure of sampling adequacy, with adequacy considered >0.6. Eigenvalue and scree-plot analysis were used to assess extracted values, and item loading on the extracted components was based on a varimax-rotated model of factor analysis. A communality cut-off value of 0.4 was considered. Internal consistency for all CMIs was tested with Cronbach’s α.

## 3. Results

The sample included 50 police working dogs of both sexes (30 males and 20 females), with a mean age of 6.5 ± 2.4 years and body weight of 26.7 ± 5.2 kg. Four breeds were represented: German shepherd dogs (*n* = 17), Belgian Malinois shepherd dogs (*n* = 15), Labrador retrievers (*n* = 10) and Dutch shepherd dogs (*n* = 8). In the six considered evaluation moments, a response for all CMIs was obtained for every animal, accounting to 300 responses for each CMI.

**Figure 1:**
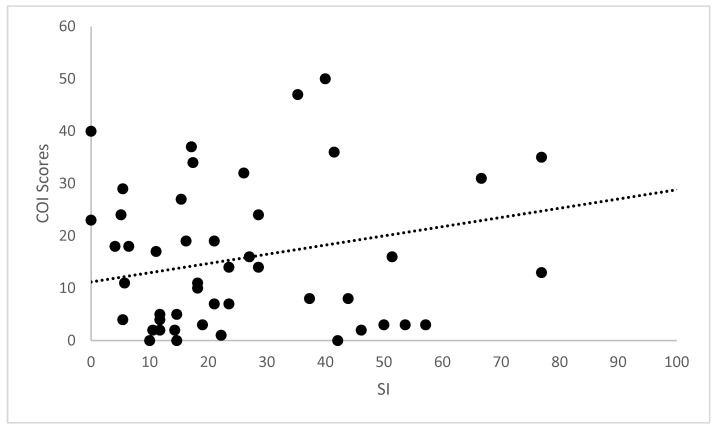
Scatterplot of COI (Canine Orthopaedic Index) versus SI (symmetry index) in the cross-sectional cohort. A significant weak correlation was observed (r = 0.21, *p* < 0.01).

**Figure 2:**
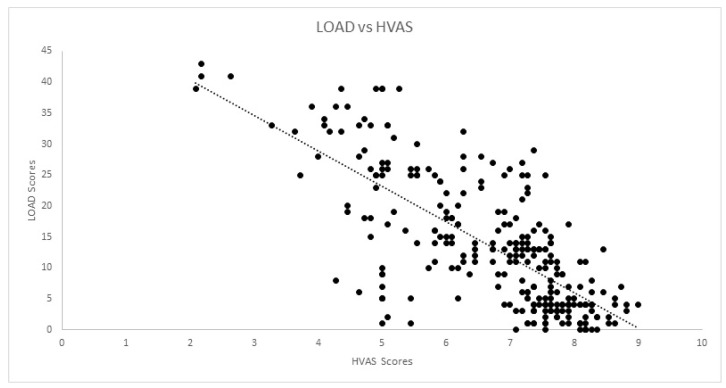
Scatterplot of LOAD (Liverpool Osteoarthritis in Dogs) and HVAS (Hudson Visual Analogue Scale) in the longitudinal cohort. A significant strong correlation was observed (r = −0.736, *p* < 0.01).

**Figure 3:**
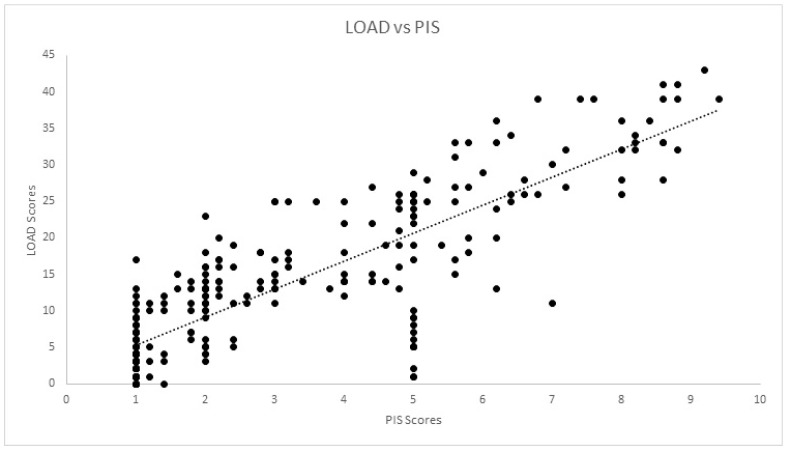
Scatterplot of LOAD (Liverpool Osteoarthritis in Dogs) and PIS (Pain Interference Score) in the longitudinal cohort. A significant strong correlation was observed (r = −0.844, *p* < 0.01).

**Figure 4:**
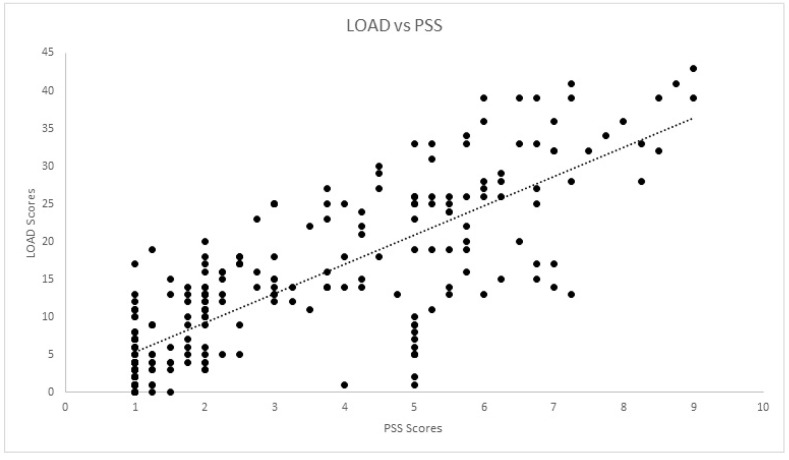
Scatterplot of LOAD (Liverpool Osteoarthritis in Dogs) and PSS (Pain Severity Score) in the longitudinal cohort. A significant strong correlation was observed (r = −0.807, *p* < 0.01).

**Figure 5:**
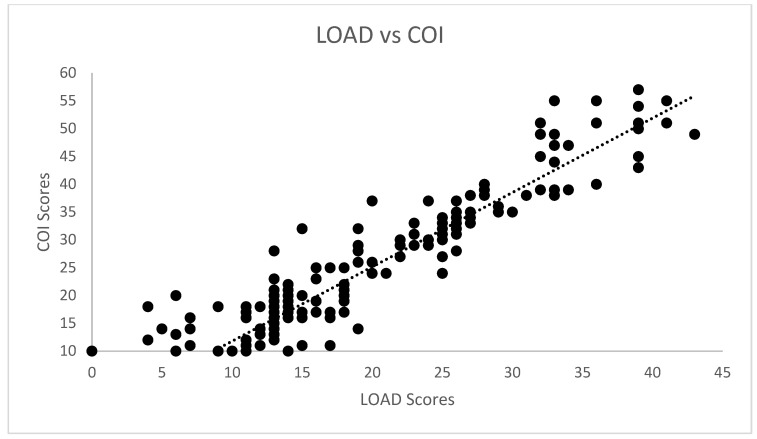
Scatterplot of LOAD (Liverpool Osteoarthritis in Dogs) and COI (Canine Orthopaedic Index) in the longitudinal cohort. A significant strong correlation was observed (r = −0.954, *p* < 0.01).

Correlations for the cross-sectional cohort are presented in Table 2. Significant strong correlation was observed between all CMIs for the cross-sectional cohort. Significant weak correlation was observed between SI and COI, and its dimensions of stiffness, function and gait. Significant moderate correlation was also observed between pedometer step count and LOAD, COI, stiffness, function, gait and QOL. The scatterplot of COI versus SI is presented in Figure 1. Correlations for the longitudinal cohort between changes of different evaluations performed are presented in Table 3. Significant strong correlations were observed between all CMIs for the longitudinal cohort. Additionally, significant weak correlations were observed between SI and all other evaluations except pedometer step count. The same was found for deviation. The scatterplots of LOAD versus HVAS, PIS, PSS and COI are presented in Figure 2, Figure 3, Figure 4 and Figure 5, respectively.

**Table 2:** Correlations for the cross-sectional cohort. COI: Canine Orthopaedic Index; HVAS: Hudson Visual Analogue Scale; LOAD: Liverpool Osteoarthritis in Dogs; PIS: Pain Interference Score; PSS: Pain Severity Score; QOL: quality of life; SI: symmetry index. *: indicates significant correlation.

Measure		SI	Deviation	Pedometer	HVAS	PIS	PSS	LOAD	COI	Stiffness	Function	Gait	QOL
SI	r_s_	1.00	0.072	−0.040	−0.203	0.036	0.196	0.300	0.324	0.026	0.367	0.324	0.177
	Sig.		0.617	0.823	0.161	0.300	0.178	0.036 *	0.023 *	0.318	0.010 *	0.023 *	0.224
Deviation	r_s_	0.072	1.00	−0.200	−0.151	0.198	0.210	0.169	0.240	0.180	0.233	0.229	0.265
	Sig.	0.617		0.256	0.200	0.174	0.147	0.245	0.096	0.216	0.107	0.114	0.066
Pedometer	r_s_	−0.040	−0.200	1.00	0.326	−0.323	−0.323	−0.425	−0.523	−0.531	−0.499	−0.427	−0.530
	Sig.	0.823	0.256		0.060	−0.063	0.078	0.012 *	0.002 *	0.000 *	0.003 *	0.012 *	00.01 *
HVAS	r_s_	−0.203	−0.151	0.326	1.00	−0.806	−0.775	−0.698	−0.643	−0.640	−0.578	−0.577	−0.652
	Sig.	0.161	0.200	0.060		0.000 *	0.000 *	0.000 *	0.000 *	0.000 *	0.000 *	0.000 *	0.000 *
PIS	r_s_	0.036	0.198	−0.323	−0.806	1.00	0.966	0.831	0.842	0.808	0.774	0.793	60.801
	Sig.	0.300	0.174	−0.063	0.000 *		0.000 *	0.000 *	0.000 *	0.000 *	0.000 *	0.000 *	0.000 *
PSS	r_s_	0.196	0.210	−0.323	−0.775	0.966	1.00	0.784	0.811	0.772	0.722	0.770	0.800
	Sig.	0.178	0.147	0.078	0.000 *	0.000 *		0.000 *	0.000 *	0.000 *	0.000 *	0.000 *	0.000 *
LOAD	r_s_	0.300	0.169	−0.425	−0.698	0.831	0.784	1.00	0.936	0.918	0.910	0.854	0.848
	Sig.	0.036	0.245	0.012 *	0.000 *	0.000 *	0.000 *		0.000 *	0.000 *	0.000 *	0.000 *	0.000 *
COI	r_s_	0.324	0.240	−0.523	−0.643	0.842	0.811	0.936	1.00	0.964	0.937	0.954	0.905
	Sig.	0.023 *	0.096	0.002 *	0.000 *	0.000 *	0.000 *	0.000 *		0.000 *	0.000 *	0.000 *	0.000 *
Stiffness	r_s_	0.026	0.180	−0.531	−0.640	0.808	0.772	0.918	0.964	1.00	0.885	0.907	0.836
	Sig.	0.318	0.216	0.001 *	0.000 *	0.000 *	0.000 *	0.000 *	0.000 *		0.000 *	0.000 *	0.000 *
Function	r_s_	0.367	0.233	−0.499	−0.578	0.774	0.722	0.910	0.937	0.885	1.00	0.830	0.810
	Sig.	0.010 *	0.107	0.003 *	0.000 *	0.000 *	0.000 *	0.000 *	0.000 *	0.000 *		0.000 *	0.000 *
Gait	r_s_	0.324	0.229	−0.427	−0.577	0.793	0.770	0.854	0.954	0.907	0.830	1.00	0.809
	Sig.	0.023 *	0.114	0.012 *	0.000 *	0.000 *	0.000 *	0.000 *	0.000 *	0.000 *	0.000 *		0.000 *
QOL	r_s_	0.177	0.265	−0.530	−0.652	0.801	0.800	0.848	0.905	0.836	0.810	0.809	1.00
	Sig.	0.224	0.066	0.01 *	0.000 *	0.000 *	0.000 *	0.000 *	0.000 *	0.000 *	0.000 *	0.000 *	

**Table 3:** Correlations for the longitudinal cohort. COI: Canine Orthopaedic Index; HVAS: Hudson Visual Analogue Scale; LOAD: Liverpool Osteoarthritis in Dogs; PIS: Pain Interference Score; PSS: Pain Severity Score; QOL: quality of life; SI: symmetry index. *: indicates significant correlation.

Measure		SI	Deviation	Pedometer	HVAS	PIS	PSS	LOAD	COI	Stiffness	Function	Gait	QOL
SI	r_s_	1.00	0.261	−0.040	−0.152	0.196	0.130	0.230	0.211	0.177	0.227	0.195	0.202
	Sig.		0.000 *	0.823	0.013 *	0.001 *	0.033 *	0.000 *	0.001 *	0.004 *	0.000 *	0.001 *	0.001 *
Deviation	r_s_	0.261	1.00	−0.200	−0.127	0.141	0.112	0.133	0.174	0.129	0.156	0.191	0.179
	Sig.	0.000 *		0.256	0.038 *	0.021 *	0.068	0.030 *	0.004 *	0.035 *	0.010 *	0.002 *	0.003 *
Pedometer	r_s_	−0.040	−0.200	1.00	0.063	−0.147	−0.118	−0.168	−0.183	−0.167	−0.260	−0.135	−0.122
	Sig.	0.823	0.256		0.412	0.055	0.125	0.028 *	0.017 *	0.029 *	0.001 *	0.078	0.111
HVAS	r_s_	−0.152	−0.127	0.063	1.00	−0.830	−0.814	−0.736	−0.716	−0.695	−0.057	−0.677	−0.732
	Sig.	0.013 *	0.038 *	0.412		0.000 *	0.000 *	0.000 *	0.000 *	0.000 *	0.355	0.000 *	0.000 *
PIS	r_s_	0.196	0.141	−0.147	−0.830	1.00	0.956	0.844	0.853	0.828	0.805	0.808	0.813
	Sig.	0.001 *	0.021 *	0.055	0.000 *		0.000 *	0.000 *	0.000 *	0.000 *	0.000 *	0.000	0.000 *
PSS	r_s_	0.130	0.112	−0.118	−0.814	0.956	1.00	0.807	0.817	0.792	0.755	0.783	0.785
	Sig.	0.033 *	0.068	0.125	0.000 *	0.000 *		0.000 *	0.000 *	0.000 *	0.000 *	0.000 *	0.000 *
LOAD	r_s_	0.230	0.133	−0.168	−0.736	0.844	0.807	1.00	0.954	0.928	0.919	0.912	0.863
	Sig.	0.000 *	0.030 *	0.028 *	0.000 *	0.000 *	*		0.000 *	0.000 *	0.000 *	0.000 *	0.000 *
COI	r_s_	0.211	0.174	−0.183	−0.716	0.853	0.817	0.954	1.00	0.966	0.952	0.965	0.916
	Sig.	0.001 *	0.004 *	0.017 *	0.000 *	0.000 *	0.000 *	0.000 *		0.000 *	0.000 *	0.000 *	0.000 *
Stiffness	r_s_	0.177	0.129	−0.167	−0.695	0.828	0.792	0.928	0.966	1.00	0.906	0.919	0.847
	Sig.	0.004 *	0.035 *	0.029 *	0.000 *	0.000 *	0.000 *	0.000 *	0.000 *		0.000 *	0.000 *	0.000 *
Function	r_s_	0.227	0.156	−0.260	−0.643	0.805	0.755	0.919	0.952	0.906	1.00	0.872	0.836
	Sig.	0.000 *	0.010 *	0.001 *	0.000 *	0.000 *	0.000 *	0.000 *	0.000 *	0.000 *		0.000 *	0.000 *
Gait	r_s_	0.195	0.191	−0.135	−0.677	0.808	0.783	0.912	0.965	0.919	0.872	1.00	0.965
	Sig.	0.001 *	0.002 *	0.078	0.000 *	0.000 *	0.000 *	0.000 *	0.000 *	0.000 *	0.000 *		0.000 *
QOL	r_s_	0.202	0.179	−0.122	−0.732	0.813	0.785	0.863	0.916	0.847	0.836	0.965	1.00
	Sig.	0.001 *	0.003 *	0.111	0.000 *	0.000 *	0.000 *	0.000 *	0.000 *	0.000 *	0.000 *	0.000 *	

**Figure 6:**
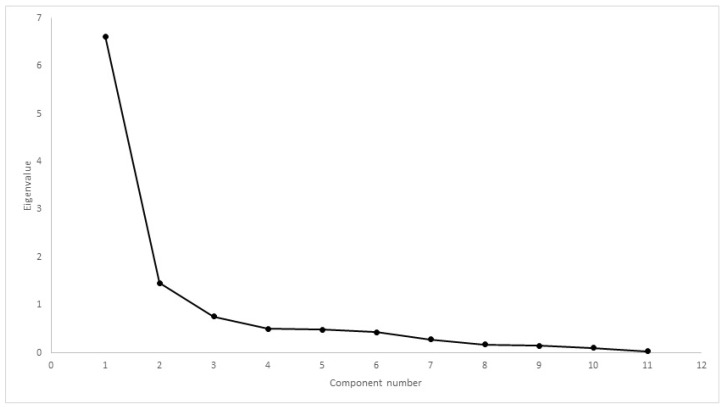
Scree plot of factor analysis of HVAS (Hudson Visual Analogue Scale). Two factors had eigenvalues >1, with a discernible “shoulder” observed.

**Figure 7:**
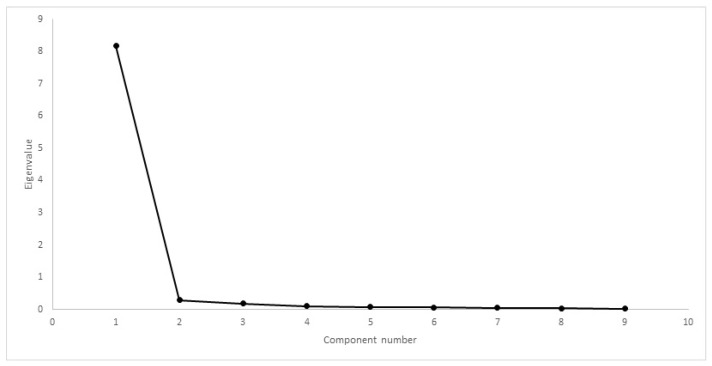
Scree plot of factor analysis of CBPI. One factor had eigenvalue >1, with a discernible “shoulder” observed.

**Figure 8:**
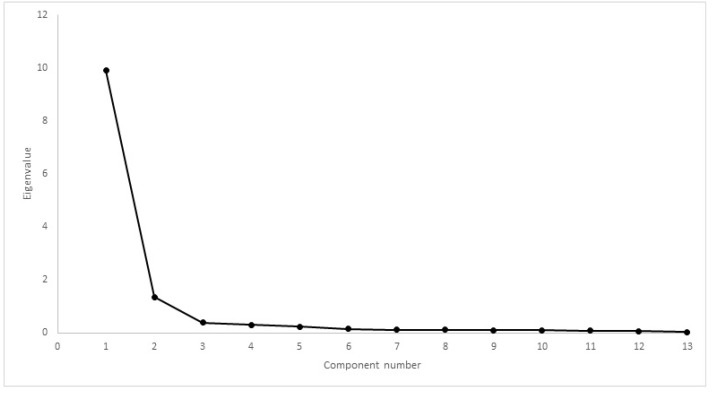
Scree plot of factor analysis of LOAD. Two factors had eigenvalues >1, with a discernible “shoulder” observed.

**Figure 9:**
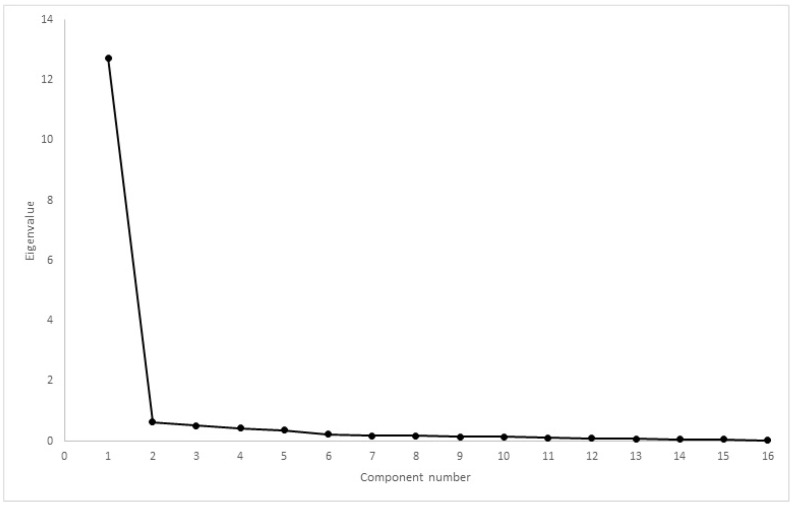
Scree plot of factor analysis of COI. One factor had eigenvalue >1, with a discernible “shoulder” observed.

Comparing CMI results, Mauchly’s test rejected the presence of sphericity (χ2(35) = 5189, *p* < 0.01). The difference between mean scores was significantly different, with F(1.18, 320,548) = 257,845, *p* < 0.01. Cronbach’s α was 0.80 for HVAS, 0.98 for CBPI, 0.97 for PIS, 0.98 for PSS, 0.97 for LOAD, 0.98 for COI, 0.97 for stiffness, 0.97 for function, 0.96 for gait and 0.85 for QOL. Kaiser–Meyer–Olkin factor analysis was 0.88 for HVAS, 0.94 for CBPI, 0.96 for LOAD and 0.96 for COI. As all values were above 0.8, factor analysis was conducted for the four CMIs. Scree plots for HVAS, CBPI, LOAD and COI are presented in Figure 6, Figure 7, Figure 8 and Figure 9, respectively. For HVAS, two factors with eigenvalues >1 were extracted. These factors accounted for 60.2% and 13.3%, respectively, of the total variance. Based on the varimax-rotated solution, loading for these items was performed. All items loaded heavily on the first component, with communalities ranging between 0.68 and 0.91. For the second component, not all items demonstrated significant communalities, with values ranging from −0.39 to 0.65. For CBPI, a single factor was extracted, which accounted for 90.9% of total variation. All other items loaded heavily on this component, with communality being >0.93. For LOAD, two factors were extracted and were responsible for 76.1% and 10.5% of the total variance, respectively. Other items loaded mainly on the first component, with communalities ranging from 0.71 to 0.94. For COI also was only one factor extracted, accounting for 79.5% of total variation. All other items loaded on this component, with communalities ranging from 0.46 to 0.89.

## 4. Discussion

A good understanding and evaluation of canine pain and its toll on daily activities is paramount to the development of treatments for chronic conditions such as OA [38,39]. To our knowledge, this is the first study to present evidence of criterion validity for the HVAS and COI. It has been described for LOAD and CBPI [19], but, for the first time, we compared the scores of these four CMIs with stance analysis and mean daily step count as an outside measure of disease. We also presented construct validity for all of the instruments, as well as their internal consistency.

The evaluation of instrument validity provides evidence that it is measuring what it is supposed to measure. One of the assessments to make is construct validity, which can be performed by testing the agreement of the instrument with a recognised “gold standard” [12,19]. A weight distribution platform, as a pressure-sensitive walkway, can provide accurate and consistent measures of weight distribution with no significant difference between devices [40]. It has been proposed that bodyweight distribution at a stance may, in fact, be an equivalent or superior measurement of pain associated with hip OA than both vertical impulse and peak vertical force [29,31]. Previous reports indicate that measures of SI are reliable indicators of clinical lameness in dogs [41]. The CBPI has been reported as being able to detect a measurable effect for individual animal assessments, with results correlating with PVF [11,19,42,43,44,45,46]. LOAD scores also show a correlation with peak vertical forces generated by a force platform [18,19], as has those of the HVAS [47]. Significant weak correlations of LOAD and CBPI with SI peak vertical force have also been described, but these dogs had OA of a single joint [19]. In the present report, all animals had bilateral disease and we used an external measure of disease weight-bearing using a stance analyser. We found a significant but weak correlation between SI and COI, stiffness, function and gait scores in the cross-sectional cohort and longitudinal cohort. In the longitudinal cohort, this significant weak correlation was observed with all CMI. The fact that the correlations found are weak is not completely unexpected, as CMIs aim to evaluate OA as a whole, from its signs to the impact it has on the animal’s life, while stance analysis is directed at evaluating function. This may explain why, in the cross-sectional cohort, correlations occur precisely with the scores that aim to evaluate more functional parameters of the disease. An additional possibility is the fact that all animals exhibited bilateral disease, which may lead to less marked asymmetries between contralateral limbs. We still chose to use this evaluation, as even a bilateral disease does not necessarily show equal signs in both limbs. Additionally, since all animals had bilateral hip OA, we performed a second analysis with the stance analyser to measure the deviation from the normal weight distribution on the pelvic limbs. This evaluation also demonstrated a significant weak correlation with all CMI scores except for PSS. Weight-bearing evaluation also has the advantage of allowing comparisons between different breeds and can still evaluate dogs that are unable to trot [19]. A second external measure of disease, which was mean daily step counts, was used. Mobility impairment and decreased activity are associated with musculoskeletal pain in humans, with improved results regarding mobility recommended as measures of outcome [17]. Pedometers have the advantage of activity monitoring over a prolonged period of time in the patient’s home environment [20,35]. In normal dogs, pedometer steps can reasonably estimate distance travelled and record data over several days, in opposition to single-day recordings [48]. We observed a significant moderate correlation with LOAD and COI in the cross-sectional cohort and a weak correlation with the same CMIs in the longitudinal cohort. The reason for a weaker correlation on the longitudinal cohort may be associated with the fact that the animals of this sample are active working dogs. This means that the overall activity of these animals is not only voluntary activity, but also varies with operational activity. For example, if one of the search and rescue dogs is engaged in a real situation and not just training during the pedometer evaluation week, it will increase the activity count. This “individual” effect may be balanced in this analysis with the fact that the work/training/rest routines are quite balanced within the population, so that the animals do not go through long periods where they kennelled or were under intense workloads. We anticipated that an increase in patient complaints (reflected in CMI scores) would correspond to higher activity levels (reflected in higher pedometer counts), and vice versa. However, a negative correlation was found, and pedometer counts followed the significant variations observed in these two CMIs scores, meaning that as activity levels (measured by the pedometer count) increase, CMI scores reduce (corresponding to better scores). A possible explanation for this may be an increase in spontaneous and playing activity, since an animal that has less pain will probably move more and be more willing to play with the handler, who is also more likely to engage in these activities, seeing the animal more active.

Construct validity can be assessed through factor analysis or by comparing the results of different instruments. Internal consistency is most frequently tested using Cronbach’s α [12,15,19]. Our results determined Cronbach’s α values > 0.90, a value that may suggest that some items are redundant and may be testing the same question in a different way [49]. This finding is in contrast with previous reports [19,22,50]. It is not surprising that Cronbach’s α varies in performance between different populations, and is possibly influenced by a homogeneous sample with a low overall variance of item scores [51]. This should be evaluated in future studies. A strong correlation was observed between CMIs in both cohorts. Previous reports have observed moderate to good correlation between LOAD and CBPI [19,52]. Each CMI was designed based on different animal populations, encompassing different breeds, sizes and OA in different joints. In addition, OA is a multidimensional disease, and different CMIs may evaluate or capture different components of the disease. Factor analysis for some of the CMIs extracted a different number of components when compared with previous reports: of LOAD, two components were extracted, compared with the three described components [19]; of CBPI, one component was extracted, similar to one report [19], but differing with the two components in another [42]; and of COI, one component was extracted, in contrast with four components in a previous study [22]. Different factor analysis with different populations is not unusual. In terms of composition, our sample is homogeneous compared with those of previous reports, with fewer breeds, similar in size and conformation, all with bilateral OA of the same joint, and with similar levels of activity. Another possible explanation may be related to the proxy completing the CMIs. It has been described that quantifying pain and attributing it a score is subjective and, therefore, may lack validity when performed by individuals unfamiliar with signs of pain [53,54]. In our study, the proxy for all dogs were experienced handlers used to observing working and sporting dogs, particularly their own, and detecting changes in movement pattern, performance losses and, possibly, changes in response to treatment. Alternative construct validity was performed through factor analysis. Factors extracted with eigenvalues greater than one or through scree-plot analysis were the same: two for HVAS and COI and one for CBPI and COI. Item loading of the components for HVAS and LOAD identified items that could be described with “ability to exercise/how often does the dog stop during exercise”, “mobility/attitude” and “stiffness/disability”. Even though CBPI and COI share some similar items with HVAS and LOAD—ability to rise or jump, stiffness and demeanour—this did not result in the extraction of more components. For CBPI, this has been described before [19]. Still, we present enough data that show that these CMIs address the clinical manifestations of OA and are able to detect changes as a result of treatment.

## 5. Conclusions

In this study, we determined criterion and construct validity of the Hudson Visual Analogue Scale, the Canine Brief Pain Inventory, the Liverpool Osteoarthritis in Dogs and the Canine Orthopaedic Index. These instruments were able to capture the various aspects of what constitutes the multi-dimensional experience that is OA. Therefore, they are valid tools to be used for the evaluation of naturally occurring canine osteoarthritis. While a strong correlation was found between the different CMIs, indicating that they all provide similarly meaningful data, recording information from more than one CMI for each patient may contribute to having a broader image of the complex experience that is OA.

## Figures and Tables

**Table 1:** Inclusion and exclusion criteria.

Inclusion Criteria	Exclusion Criteria
Mobility impairment, as described by the trainer and detected by the assisting veterinarian;	Suspected or diagnosed neurological/musculoskeletal disorder other than hip OA;
Bodyweight ≥ 15 kg;	Documented or suspected presence of concomitant disease;
Age ≥ 1 year;	Receiving any other drugs;
Radiographic evidence of bilateral OA of the hip joint;	Results of routine blood testing outside normal limits.
Not to be on any medication or nutritional supplements for six weeks or more.	

## Data Availability

All data generated or analysed during this study are included in this published article.
